# Salivary uric acid across child development and associations with weight, height, and body mass index

**DOI:** 10.3389/fped.2023.1235143

**Published:** 2023-11-01

**Authors:** J. L. Riis, A. L. Dent, O. Silke, D. A. Granger

**Affiliations:** ^1^ Department of Kinesiology and Community Health, University of Illinois at Urbana-Champaign, Urbana, IL, United States; ^2^ Institute for Interdisciplinary Salivary Bioscience Research, University of California, Irvine, CA, United States; ^3^ Department of Psychological Science, University of California, Irvine, CA, United States; ^4^ Department of Pediatrics, Johns Hopkins University School of Medicine, Baltimore, MD, United States

**Keywords:** uric acid, saliva, obesity, body mass index, child, Family Life Project

## Abstract

**Introduction:**

Obesity during childhood is a serious and growing chronic disease with consequences for lifelong health. In an effort to advance research into the preclinical indicators of pediatric obesity, we examined longitudinal assessments of uric acid concentrations in saliva among a cohort of healthy children from age 6-months to 12-years (*n*'s per assessment range from 294 to 727).

**Methods:**

Using data from a subsample of participants from the Family Life Project (an Environmental influences on Child Health Outcomes Program cohort), we: (1) characterized salivary uric acid (sUA) concentrations from infancy to early adolescence by sex and race; (2) assessed changes in sUA levels across development; and (3) evaluated associations between sUA concentrations and measures of child weight, height, and body mass index (BMI). Across four assessments conducted at 6-, 24-, 90-, and 154-months of age, 2,000 saliva samples were assayed for UA from 781 participants (217 participants had sUA data at all assessments).

**Results:**

There were no significant differences in sUA concentrations by sex at any assessment, and differences in sUA concentrations between White and non-White children varied by age. At the 90- and 154-month assessments, sUA concentrations were positively correlated with measures of child weight, height, and BMI (90-month: weight- *ρ*(610) = 0.13, *p *< 0.01; height- *ρ*(607) = 0.10, *p *< 0.05; BMI- *ρ*(604) = 0.13, *p *< 0.01; 154-month: weight- *ρ*(723) = 0.18, *p *< 0.0001; height- *ρ*(721) = 0.10, *p *< 0.01; BMI- *ρ*(721) = 0.17, *p *< 0.0001). Group based trajectory modeling identified two groups of children in our sample with distinct patterns of sUA developmental change. The majority (72%) of participants showed no significant changes in sUA across time (“Stable” group), while 28% showed increases in sUA across childhood with steep increases from the 90- to 154-month assessments (“Increasing” group). Children in the Increasing group exhibited higher sUA concentrations at all assessments (6-month: *t*(215) = −5.71, *p *< 0.001; 24-month: *t*(215) = −2.89, *p *< 0.01; 90-month: *t*(215) = −3.89, *p *< 0.001; 154-month: *t*(215) = −19.28, *p *< 0.001) and higher weight at the 24- and 90-month assessments (24-month: *t*(214) = −2.37, *p *< 0.05; 90-month: *t*(214) = −2.73, *p *< 0.01).

**Discussion:**

Our findings support the potential utility of sUA as a novel, minimally-invasive biomarker that may help advance understanding of the mechanisms underlying obesity as well as further surveillance and monitoring efforts for pediatric obesity on a large-scale.

## 1. Introduction

Pediatric obesity is one of the most pressing public health concerns in the United States (1). Obesity affects nearly one of every five American children and rates have increased in recent years, aggravated by the COVID-19 pandemic (1–3). Obesity has serious short- and long-term health comorbidities including increased risk of developing cardiometabolic, respiratory, sleep, and psychological health conditions during childhood and into adulthood (4–7). The effects of childhood obesity on population health are further magnified by socioeconomic, racial, and ethnic disparities in prevalence rates (4). These disparities, along with obesity's lasting biological, psychological, and behavioral effects, make pediatric obesity a significant factor affecting not only child health, but also propelling health disparities in the US (1, 4, 6). However, obesity, and many of its comorbidities, during childhood, is reversable with targeted health behavior and clinical interventions (1).

Despite decades of research into the development and perpetuation of obesity across the lifespan, our ability to develop effective programs to prevent pediatric obesity remain limited (5, 8–10). Key to these efforts is the need to identify children at risk of obesity, as well as to further our understanding of the mechanisms by which obesity increases risk of poor health outcomes in children and adults (1). Identifying biological indicators of obesity risk before disease onset could allow for early interventions targeting change in the biopsychosocial pathways leading to obesity and its comorbidities. To address these needs, the current study examines uric acid (UA), an analyte with well characterized associations with obesity and cardiometabolic disease risk, prospectively in salivary biospecimens collected from a cohort of infants/children from ages 6-months to 12-years. We examine the child- and family-level correlates of salivary UA (sUA) measurements in this sample with the goal of elucidating the utility of sUA as a potential early indicator of risk for pediatric obesity.

### 1.1. Uric acid and obesity

UA in the body can be derived from both exogenous (e.g., diet) and endogenous sources (11). It has several physiologic functions including activating the renin-angiotensin system and mediating and amplifying the type 2 inflammatory response (11, 12). In the cardiovascular system, UA can play a key role in vascular restriction (11, 13). Given its multiple effects and functions, elevated levels of UA in serum are associated with increased risk of metabolic health indicators, including risk of obesity, metabolic syndrome, hypertension, diabetes, inflammation, and cardiovascular disease among adults, adolescents, and children (14, 15, 24–28, 16–23). The link between high levels of UA and obesity may be explained by many factors including its effects on adipose tissue, which can lead to oxidative stress, inflammation, and reduced adiponectin levels, as well as its effects on the liver, which can contribute to oxidative stress, fat accumulation, and insulin resistance (12).

While the vast majority of prior research examining the associations between UA and obesity and cardiometabolic risk have been conducted using serum, the serum-saliva correlation for UA is strong (29–31), and levels in saliva have been shown to be relatively stable among adults (29). As with concentrations in serum, UA levels in saliva show positive associations with cardiometabolic risk indicators, such as blood pressure and BMI (32, 33). The ability to reliably assess UA concentrations in saliva opens up additional research and practice opportunities to evaluate UA longitudinally in younger, even infant, populations. Advancing our understanding of sUA and its associations with health risks across the lifespan is an important step to developing the utility of UA as a potential early indicator of obesity and its comorbidities.

### 1.2. The present study

To our knowledge, this study is the first large-scale, longitudinal investigation of sUA in healthy children and the first examination of the developmental trajectory of sUA from infancy to adolescence. Currently, there are no standard reference ranges for sUA in the pediatric population. We aim to generate new information about the levels and correlates of sUA from infancy to early adolescence and assess the extent to which sUA levels are associated with BMI and obesity risk. Specifically, we examined resting sUA concentrations among children ages 6-months to 12-years to: 1) characterize sUA levels across infancy/childhood by sex and race in our sample; 2) assess changes in sUA concentrations across development; and 3) evaluate associations between sUA concentrations and child weight, height, and BMI. Based on prior studies, we hypothesized that concentrations of sUA would increase with age (20, 34) and display positive associations with indices of overweight/obesity, especially at older ages (24, 25, 34). We also hypothesized that, at older ages, concentrations of sUA would be higher among male compared to female children (20, 24, 29, 34).

## 2. Methods

### 2.1. Sample and procedures

This is a secondary data analysis study that uses data from the Family Life Project (FLP). FLP is an ongoing, population-based, longitudinal cohort study of child health and development in the context of rural poverty and an Environmental influences on Child Health Outcomes (ECHO) Program cohort. In 2003, FLP recruited a representative sample of 1,292 infants residing in six counties (three in Pennsylvania (PA), three in North Carolina (NC)) at birth. Low-income families in both states and African American families in NC were oversampled. African Americans were not oversampled in PA, as the target communities were 95+% non-African American. Data collection from children and caregivers occurred in participants' homes, or over the phone, from child age 2-months to 12 years (and ongoing). Attrition in the sample has been low; 2% over the first three years and 11% through 3rd grade. Detailed descriptions of the FLP sampling strategy have been previously published (35).

#### 2.1.1. Sample

This study uses data from the 6-month, 24-month, 90-month (approximately 7 years old), and 154-month (approximately 12 years old) FLP cohort study assessments. The subsamples of children evaluated in this study were selected based on the availability of archived salivary biospecimens for the 6- and 24-month samples, the assaying of these samples for sUA, and existing data. Since archived biospecimens were used to generate sUA data for the earliest two timepoints, sample sizes for these assessments were smaller than those at the 90- and 154-month assessments (Supplementary Table S1).

#### 2.1.2. Saliva collection procedures

The UA data used in this study come from unstimulated salivary biospecimens collected from child participants at each of the four FLP study assessments of interest. All biospecimens were collected as resting/baseline samples meaning that they were collected prior to the initiation of study specific challenge tasks and in the absence of any specific stimuli imposed upon the participant as part of the research study. At the 6- and 24-month assessments, saliva was collected using absorbent swabs. At the 90- and 154-month assessments, saliva was collected using the passive drool technique. All saliva samples were archived at −80°C at the University of California, Irvine's Institute for Interdisciplinary Salivary Bioscience Research (IISBR).

### 2.2. Measures

#### 2.2.1. Salivary uric acid (sUA)

Following Riis and colleagues (29), UA was assayed in duplicate using a commercially-available, enzymatic assay kit (Salimetrics; Item# 1-3802) specifically designed for use with oral fluid. The test volume was 10 µl, and the lower and upper limits of sensitivity for this assay are 0.07 mg/dl and 20 mg/dl, respectively. sUA concentrations were calculated from a standard proportion in which the kit manufacturer has established linearity from 0 to 20 mg/dl (Gen5, BioTek). The intra- and inter-assay coefficients of variation (CV) were 3.98 and 3.10%, respectively (intra-assay CVs by assessment time: 6-months- 4.74%; 24-months- 4.43%; 90-months- 4.24%; 154-months- 3.15%).

#### 2.2.2. Child demographics

Levels of sUA were examined by child sex (male/female), age (months), and caregiver-reported race. Child race was dichotomized as White vs. non-White due to the distribution of race in the sample (Table 1, Supplementary Table S1).

**Table 1:** Characteristics of child participants with complete salivary uric acid data at all four assessments (*n* = 217).

	*M*	*SD*	Min	Max	*n*
Male (*n*, %)	121	56%			217
Race (*n*,%)					217
White	139	64%			
Black/African American	78	36%			
American Indian or Alaska Native	0	0%			
Asian	0	0%			
Hispanic/Latino (*n*, %)	7	3%			217
State (*n*, %)					217
North Carolina	90	41%			
Pennsylvania	127	59%			
Family income/needs	2.09	1.93	0.00	16.47	201
Caregiver married (*n*, %)	115	53%			217
Caregiver education (years)	14.82	2.69	8	22	217

All data were reported by caregivers at the 6-month study assessment. See Supplementary Table S1 for characteristics of the sample at each assessment time. *M*, mean; *SD*, standard deviation; Min, minimum; Max, maximum.

#### 2.2.3. Child weight, height, and body mass index

Child weight-for-age percentile, length-, or height-, for-age percentile, and BMI-for-age percentile scores were created using measurements conducted by trained research staff at each study visit. BMI was not calculated for the 6-month visit data nor for participants younger than 23.5 months old at the 24-month assessment (1). BMI-for-age percentile scores were examined as a continuous variable, and data were also dichotomized to compare children with overweight/obesity to children who were underweight or of a healthy weight using the Centers for Disease Control and Prevention's age- and sex-specific BMI criteria which categorizes children with BMIs greater than or equal to the 85th percentile with overweight/obesity and children with BMIs less than the 85th percentile with non-overweight/obesity (36).

#### 2.2.4. Covariates

Child and family characteristics with potential associations with sUA concentrations were examined at each assessment time. Child-level covariates included birth weight [ounces (37)], ethnicity (Hispanic/Latino vs. non-Hispanic/Latino), recent medication use (yes/no; see Supplemental Methods for details), recent food/drink intake (ate/drank in the last 60-minutes- yes/no; not available at the 24-month assessment), current health (i.e., relative to other children of the same age, health in past 2 days has been: excellent/very good/good/fair/poor; fair and poor response categories were collapsed for analysis purposes (Supplementary Table S1; not available at the 24-month assessment)), and teething status (yes/no; available at the 6-month assessment only). Family-level factors included primary respondent's age (years), education (years), and marital and employment status (married/not married; employed/not employed) at the 6-month assessment, and family income-to-needs ratio. Study visit timing (time since noon; minutes) was also examined given the diurnal rhythm of sUA concentrations (38–42). All covariate data were caregiver-reported except health status, medication use, and recent food/drink intake at the 154-month assessment were self-reported by adolescents, and saliva sample timing data were recorded by research staff.

### 2.3. Statistical analyses

#### 2.3.1. Preanalytical processing of salivary uric acid data

The nature and distribution of sUA data were examined at each assessment time. sUA concentration data for samples with sUA levels too low to be reliably measured by the assay were replaced with half the assay's lower limit of detection for analysis purposes (0.035 mg/dl (43, 44)).

#### 2.3.2. Salivary uric acid concentrations by assessment time

sUA concentrations from infancy to early adolescence were examined using descriptive statistics. Differences in sUA levels by sex and race were examined at each assessment using Wilcoxon rank-sum tests. Bivariate tests of association were used to examine the correlates of sUA concentrations at each assessment time, including the effects of potential covariates of sUA levels, and the relations between sUA concentrations and measures of child weight, height, and BMI (e.g., using Spearman correlation, Kruskal-Wallis and Wilcoxon Rank Sum tests, and ANOVA).

Adjusted relations between sUA concentrations and child weight-for-age, height-for-age, and BMI-for-age percentile scores, as well as differences by race and sex, were estimated using multivariable linear regression models for sUA concentrations at each assessment time separately. These models accounted for the effects of child age, sex, and race, family income-to-needs ratio, the timing of the study visit, and other covariates showing statistically significant associations with sUA levels in bivariate analyses. Model fit was evaluated for all linear regression models using residual plots, and influential cases affecting model fit were excluded.

#### 2.3.3. Developmental changes in salivary uric acid concentrations

Group-based trajectory modeling [GBTM; (45, 46)] was used to identify groups of children whose developmental trajectories of sUA followed similar patterns across time. Model selection followed a three-step process. First, we identified the appropriate number of trajectory groups using a stepwise approach, examining models with one to four trajectory groups. At this stage, no covariates were included in the model. Second, polynomial functions for time were evaluated for each trajectory group by first specifying a quadratic shape and moving down polynomial specifications if the quadratic shape was not significant. Third, child age at assessment was added as a time-dependent covariate to the best fitting model, and polynomial shapes for each trajectory group were re-evaluated and adjusted as needed.

Time was rescaled so that the 6-month assessment represented the intercept. Model fit was evaluated using several criteria including Bayesian information criterion (BIC), Adjusted BIC, Akaike information criterion (AIC), and entropy (47). These criteria, as well as a consideration of the interpretability of group size and meaningfulness of differences in trajectories, were used to select the final trajectory model. In addition, we examined the average posterior probability and the odds of correct classification (based on the weighted posterior probability) to assess model performance [using thresholds of >.70 and ≥5.0, respectively (47, 48)].

Differences in sUA concentrations and child and family characteristics, including sociodemographic characteristics and measures of child weight, height, and BMI, across sUA developmental trajectory groups were examined using independent means *t*-tests, Rank Sum tests, and chi-squared tests.

Analyses conducted using data from a single assessment time used all available sUA data from that timepoint while trajectory model analyses were restricted to those participants with complete sUA data across all four assessments (*n *= 217; Table 1, Supplementary Figure S1). All analyses were conducted using complete case analysis. To address missingness in the independent variables, sensitivity analyses were conducted for all linear regression models using multiple imputation by chained equations generating 25 datasets and pooling results across repeat analyses. Also, given that missingness in BMI and height-for-age percentile data were related to child age at the 24-month assessment (with no percentile data available for children younger than 23.5-months old), a set of sensitivity analyses examined associations between sUA measures and child height (in cm, rather than percentile scores) at the 24-month visit. Tests of statistical significance were two-sided with an alpha of 0.05, and Bonferroni-corrected alpha levels (0.05/the number of tests) were also examined when evaluating associations between sUA concentrations and indices of child weight, height, and BMI. All analyses were conducted using Stata (versions 17.0 and 15.1 [*traj* package]; StataCorp LP, College Station, Texas).

## 3. Results

### 3.1. Data and sample characteristics

**Figure 1:**
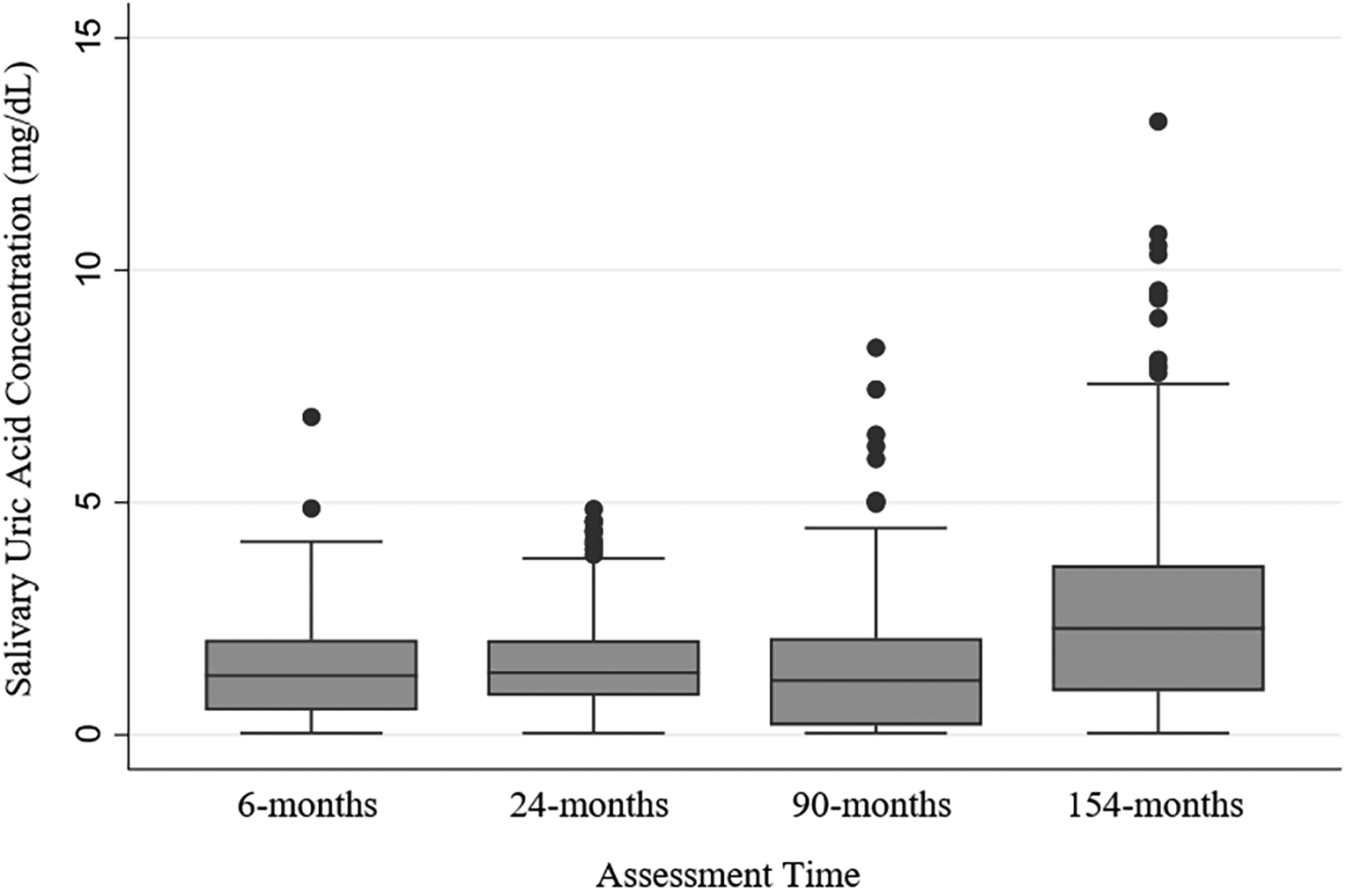
Salivary uric acid concentrations (mg/dl) among healthy children by assessment time. *N*'s by assessment time: 6-months = 362; 24-months = 294; 90-months = 616; 154-months = 727; *N*'s by assessment time are overlapping with 781 participants sampled repeatedly across the assessments, and 217 participants with salivary uric acid data at every assessment (see Supplementary Figure S1 for additional information about samples across assessments).

Across all assessments, 2,000 saliva samples were assayed for UA from 781 participants. The vast majority of saliva samples (91%; *n *= 1,825) were tested with minimal measurement and reliability issues. Eight percent of the saliva samples tested (*n *= 153) had concentrations of sUA that were too low to be reliably measured with the assay (i.e., <0.07 mg/dl), and these data were replaced with half the assay's lower limit of detection (43, 44). sUA data were moderately normally distributed at each assessment time (skew and kurtosis by assessment time: 6-months- 0.93, 4.69; 24-months- 0.94, 3.94; 90-months- 1.25, 5.33; 154-months- 1.12, 5.41; Figure 1).

Of the 781 participants with sUA data evaluated for this study, 217 had sUA data at every assessment; 136 were missing sUA data from one assessment; 295 were missing sUA data at two assessments; and 133 were missing data from three assessments (see Supplementary Figure S1 for additional information regarding sample sizes and the degree of overlap in samples across assessments). Table 1 shows the characteristics of the subsample of children with complete sUA data at all assessments (*n *= 217). Characteristics of the samples of children with sUA data at each assessment are provided in Supplementary Table S1. Our sample was moderately well balanced for child sex and state of residence, and approximately half of child participants had caregivers who were married at the 6-month assessment. The sample was approximately 60% White and the majority of the remaining children were identified as Black/African American by their caregivers. At the 6-month assessment, children ranged in age from 5.09 to 13.11 months (mean(*M*)(standard deviation((*SD*))= 7.43(1.34)); at the 24-month assessment, children were between 22.24 and 34.76 months old (*M*(*SD*)= 24.42(1.59)); at the 90-month assessment, children were between 78.85 and 100.73 months old (*M*(*SD*)= 87.03(3.27)); and at the 154-month assessment, children ranged from 150.28 to 182.08 months old (*M*(*SD*)= 158.15 (6.65)) (Supplementary Table S1). The average BMI percentile score at each assessment was within the healthy range (i.e., 5th to <85th percentile (36)), and there was a wide range of percentile scores for height, weight, and BMI at each assessment (Supplementary Table S1). Compared to the larger sample of FLP child participants, the subsample of participants with complete sUA data at all assessments used in this study (*n *= 217) included more children from PA (*χ*^2^(1)= 36.56, *p *< 0.001), more White children (*χ*^2^(1) = 5.40, *p *< 0.05), and more children from families with caregivers with higher education (*t*(1,199) = −2.20, *p *< 0.05).

There were minimal missing data for most child- and family-level covariates examined. For the subsample of participants with sUA data at the 6-month assessment (*n *= 362), the percentage of missing data for the child- and family-level covariates was ≤1% for all variables except family income-to-needs ratio data were missing for 8% of this subsample. All covariates examined among the 24-month assessment subsample (*n *= 294) had <9% missing data except recent medication use was missing for 26% of this subsample, and child height and BMI percentile scores were missing for 26% and 51% of this subsample, respectively. For the subsample of participants with sUA data at the 90-month assessment (*n *= 616), there were <5% missing data for all covariates except information about eating/drinking before the study visit were missing for 51% of this subsample. Missing data for the 154-month subsample (*n *= 727) were <3% for all covariates.

### 3.2. Salivary uric acid concentrations by assessment time

#### 3.2.1. Covariates of salivary uric acid concentrations

At some assessments, recent eating/drinking and time since noon emerged as significant covariates of sUA concentrations (recent eating/drinking at the 6-month assessment: *z *= 2.28, *p *< 0.05; *M*_ate/drank_(*SD*_ate/drank_) = 1.30(0.94); *M*_did not eat/drink_(*SD*_did not eat/drink_) = 1.56(1.09); at the 90-month assessment: *z *= −3.14, *p *< 0.01; *M*_ate/drank_ (*SD*_ate/drank_) = 1.61(1.25); *M*_did not eat/drink_(*SD*_did not eat/drink_) = 1.22(1.19); time since noon at the 24-month assessment: *ρ*(268) = −0.23, *p *< 0.001; at the 90-month assessment: *ρ*(613) = −0.07, *p *= 0.06). At the 6-month assessment, older infants exhibited lower concentrations of sUA (*ρ*(360) = −0.11, *p *< 0.05), and birthweight was positively associated with sUA levels (*ρ*(359) = 0.16, *p *< 0.01). At the 154-month assessment, sUA concentrations were highest among children with fair/poor self-reported health (*χ*^2^(3) = 11.55, *p *< 0.01). sUA concentrations did not significantly differ between teething vs. not teething infants nor between children with recent medication use vs. those without recent use at any assessment time. Hispanic/Latino children exhibited levels of sUA similar to non-Hispanic/Latino children at all assessments. Concentrations of sUA were also not significantly associated with family income-to-needs ratio, nor caregiver age, marital or employment status, nor education level.

#### 3.2.2. Salivary uric acid concentrations by sex and race

Descriptive statistics for sUA concentrations by assessment time and sex are presented in Table 2, and concentrations by assessment time and race category are presented in Supplementary Table S2. sUA concentrations were similar for male and female children at all assessments. At the 6- and 154-month assessments, sUA concentrations were different when comparing White to non-White children (6-month assessment: *z*(360) = −2.28, *p *< 0.05; *M*_White_(*SD*_White_) = 1.49(0.97), *M*_non−White_(*SD*_non−White_) = 1.30(1.08); 154-month assessment: *z*(725)= 2.34, *p *< 0.05; *M*_White_(*SD*_White_) = 2.37(2.07), *M*_non−White_(*SD*_non−White_) = 2.60(1.76)). No statistically significant differences were observed between sUA concentrations among White vs. non-White children at the 24- and 90-month assessments.

**Table 2:** Salivary uric acid concentrations (mg/dl) from infancy to early adolescence among healthy male and female children*.*

	Assessment time							
	6-months		24-months		90-months		154-months	
	Male	Female	Male	Female	Male	Female	Male	Female
Mean	1.41	1.43	1.56	1.44	1.37	1.35	2.51	2.44
*SD*	1.01	1.02	0.99	0.89	1.30	1.23	2.06	1.82
Median	1.25	1.29	1.39	1.29	1.17	1.17	2.33	2.28
Min	0.04	0.04	0.04	0.04	0.04	0.04	0.04	0.04
Max	4.16	6.84	4.86	4.58	8.33	6.46	10.52	13.20
*n*	201	161	161	133	321	295	363	364

Samples at each assessment are overlapping. See text and Supplementary Figure S1 for information about the degree of overlap and follow-up across assessments. There were no statistically significant differences in salivary uric acid (sUA) concentrations by sex at any assessment. Concentrations of sUA that were too low to be measured by the assay were replaced with 0.035 mg/dl. *SD,* standard deviation; Min, minimum; Max, maximum.

Supplementary Table S3 presents the results from adjusted models for sUA concentrations at each assessment time. After adjusting for child age, sex, recent health (not included in the 24-month model due to missingness), recent food/drink intake (not included in the 24- and 90-month models due to missingness), family income-to-needs ratio, timing of the study visit, and birthweight (6-month model only), differences in sUA concentrations between White and non-White children remained statistically significant at both the 6- and 154-month assessments. After adjusting for all covariates, age and birthweight were not significantly associated with sUA levels at the 6-month assessment, but family income-to-needs ratio was inversely related to infant sUA concentrations. At the 90-month assessment, older participants exhibited lower concentrations of sUA in the adjusted model. However, the opposite effect was observed in the adjusted model for sUA concentrations at the 154-month assessment where older participants exhibited higher sUA concentrations.

#### 3.2.3. Salivary uric acid concentrations and child weight, height, and body mass index

At the 90- and 154-month assessments, sUA concentrations were positively correlated with measures of weight, height, and BMI (Table 3) and were higher among children/adolescents with overweight/obesity compared to children/adolescents with non-overweight/obesity (at the 90-month assessment: *t*(604) = −2.49, *p *< 0.05; at the 154-month assessment: *t*(721) = −3.54, *p *< 0.001). Weight, height, and BMI measures at the 90- and 154-month assessments remained positively associated with sUA concentrations after adjusting for child age, sex, race, recent health and food/drink intake (not included in the 90-month model due to missingness), family income-to-needs ratio, and time since noon (at the 90-month assessment- weight percentile: *b *= 0.005, robust *SE *= 0.002, *p *< 0.01, 95% CI [0.002, 0.009], height percentile: *b *= 0.004, robust *SE *= 0.002, *p *< 0.01, 95% CI [0.001, 0.008]; BMI percentile: *b *= 0.005, robust *SE *= 0.002, *p *< 0.01, 95% CI [0.002, 0.009]; at the 154-month assessment- weight percentile: *b *= 0.01, robust *SE *= 0.002, *p *< 0.001, 95% CI [0.006, 0.016], height percentile: *b *= 0.008, robust *SE *= 0.002, *p *< 0.01, 95% CI [0.004, 0.013], BMI percentile: *b *= 0.01, robust *SE *= 0.003, *p *< 0.001, 95% CI [0.005, 0.015]). After adjusting for the covariates, children with overweight/obesity at the latter two assessments showed higher sUA concentrations than children with non-overweight/obesity (at the 90-month assessment: *b *= 0.25, robust *SE *= 0.11, *p *< 0.05, 95% CI [0.03, 0.48]; at the 154-month assessment: *b *= 0.38, robust *SE *= 0.14, *p *< 0.01, 95% CI [0.10, 0.66]). The adjusted associations between indices of weight, height, and BMI and sUA concentrations at the 90- and 154-month assessments were robust to corrections for multiple comparisons (Bonferroni-corrected *α*-level = 0.0125; except the difference in sUA levels between children with non-overweight/obesity and children with overweight/obesity at 90-months). No statistically significant associations between sUA concentrations and weight, height, nor BMI measures emerged in adjusted linear regression models for sUA at the 6- and 24-month assessments.

**Table 3:** Correlations between salivary uric acid concentrations (mg/dl) and measures of weight, height, and body mass index (BMI) at each assessment time among healthy children.

Assessment time	Weight-for-age percentile		Height-for-age percentile		BMI-for-age percentile	
	*ρ*	*n*	*ρ*	*n*	*ρ*	*n*
6-months	0.08	360	0.04	361		
24-months	−0.08	292	−0.05	218	−0.13	143
90-months	0.13**^,a^	612	0.10*	609	0.13**^,a^	606
154-months	0.18***^,a^	725	0.10**^,a^	723	0.17***^,a^	723

Samples at each assessment are overlapping. See text and Supplementary Figure S1 for information about the degree of overlap and follow-up across assessments. BMI was not calculated for infants.

* *p *< 0.05.

** *p *< 0.01.

*** *p *< 0.0001.

^a^ Statistically significant at the Bonferroni-corrected *α*-level (6-month assessment corrected *α* = 0.025; 24-, 90-, 154-month assessment corrected *α* = 0.0125).

### 3.3. Developmental changes in salivary uric acid concentrations

**Figure 2:**
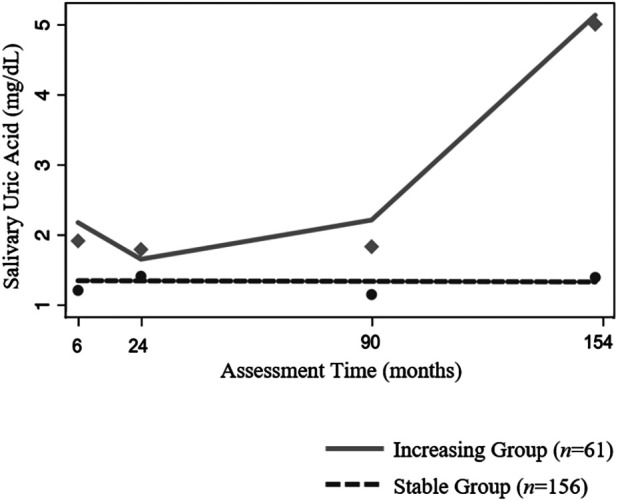
Patterns of change in salivary uric acid concentrations (mg/dl) across development among healthy children (*n = 217*). Model is adjusted for child age at assessment as a time-dependent covariate.

The best fitting GBTM, adjusting for age at assessment, indicated two patterns of developmental change in sUA in our sample (see Table 4 for fit indices for models specifying one to four groups). One group was comprised of the majority of children (72%; *n *= 156) and exhibited no statistically significant change in sUA concentrations across development (i.e., the “Stable” group; Figure 2; intercept estimate: 1.27, *SE *= 0.07, *t *= 17.62, *p < *0.001). The other group (i.e., the “Increasing” group) consisted of nearly thirty percent of the children (28%; *n *= 61) and exhibited increases in sUA concentrations from toddlerhood to adolescence with a sharp increase in sUA concentrations after the 90-month assessment (Figure 2; quadratic time parameter estimate: 0.00, *SE *= 0.00, *t *= 9.51, *p *< 0.001). At each assessment time, children in the Increasing group had, on average, higher concentrations of sUA than children in the Stable group (Figure 2; 6-month assessment: *t*(215)= −5.71, *p *< 0.001; 24-month assessment: *t*(215) = −2.89, *p *< 0.01; 90-month assessment: *t*(215) = −3.89, *p *< 0.001; 154-month assessment: *t*(215) = −19.28, *p *< 0.001). Child age at the 6-month assessment was significantly associated with sUA concentrations among children in the Increasing group (coefficient estimate: −0.06, *SE *= 0.02, *t *= −2.65, *p *< 0.01) such that those who were older at the 6-month assessment had lower sUA levels. The average posterior probability for both groups was ≥.90, and the odds of correct classification were ≥10.46 indicating high accuracy in GBT assignments (47).

**Table 4:** Model fit indices for group-based trajectory models of salivary uric acid concentrations across infancy to early adolescence with 1–4 trajectory groups.

Number of trajectory groups	Log-likelihood	AIC	BIC	BIC_ADJ_	Entropy	Size of smallest group
1	−1,523.85	−1,527.85	−1,537.39	−1,534.61	–	–
2	−1,428.58	−1,434.58	−1,448.88	−1,444.72	.82	*n* = 59
2^a^	−1,425.41	−1,433.41	−1,452.48	−1,446.93	.81	*n* = 61
3	−1,400.03	−1,410.03	−1,433.86	−1,426.93	.85	*n* = 7
4^b^	−1,384.43	−1,400.43	−1,438.56	−1,427.47	.87	*n* = 7

The polynomial structure was quadratic for the one-group model; intercept + quadratic for the two-group models; intercept + quadratic + quadratic for the three-group model; quadratic + quadratic + quadratic + quadratic for the four-group model.

^a^ Model includes child age at assessment as a time-dependent covariate.

^b^ Model results are not robust to the removal of one participant with a high salivary uric acid concentration.

#### 3.3.1. Differences in participant characteristics across trajectory groups

There was no evidence that child sex, race, or age, nor caregiver marital status or education level, varied across sUA trajectory groups. Compared to children in the Stable sUA group, on average, children in the Increasing sUA group came from higher income families (relative to needs; significant differences at all assessments except the 6-month assessment; 24-month assessment: *z *= −2.12, *p *< 0.05; 90-month assessment: *z *= −2.58, *p *< 0.05; 154-month assessment: *z *= −2.59, *p *< 0.01). Self-reported health at the 154-month assessment was also associated with trajectory group membership with a higher proportion of participants reporting fair/poor health in the Increasing sUA group (*χ*^2^(3) = 7.95, *p *< 0.05) at this timepoint. A similar, yet not statistically significant, trend was observed at the 90-month assessment (*χ*^2^(3) = 6.91, *p *= 0.08).

#### 3.3.2. Differences in child weight, height, and body mass index across trajectory groups

Supplementary Table S4 presents weight, height, and BMI data for children in each trajectory group. Compared to children in the Stable sUA group, children in the Increasing sUA group had, on average, higher weight-for-age percentile scores at the 24- and 90-month assessments and a higher BMI-for-age percentile score at the 90-month assessment (at 24-month assessment: *t*(214) = −2.37, *p *< 0.05, *M*_Increasing Group_(*SD*_Increasing Group_) = 65.16(29.44), *M*_Stable Group_(*SD*_Stable Group_) = 54.69(28.98); at 90-month assessment: weight-for-age—*t*(214) = −2.73, *p < *0.01, *M*_Increasing Group_(*SD*_Increasing Group_) = 74.76(26.77), *M*_Stable Group_(*SD*_Stable Group_) = 62.66 (30.25), BMI-for-age—*t*(212)= −2.54, *p *< 0.05, *M*_Increasing Group_(*SD*_Increasing Group_) = 72.90(27.37), *M*_Stable Group_(*SD*_Stable Group_) = 61.69(29.88)). A similar trend was observed at the 154-month assessment, with higher average weight, height, and BMI-for-age percentile scores for the Increasing sUA group compared to the Stable sUA group, however, differences between the groups on these measures were only marginally statistically significant (*p*-values = 0.05–0.10). In addition, children in the Increasing sUA group were more likely to have overweight/obesity at the 90-month assessment than children in the Stable sUA group (*χ*^2^(1) = 6.85, *p *< 0.01). Within each trajectory group, there were no statistically significant associations between sUA concentrations and measures of child weight, height, or BMI at any assessment time.

### 3.4. Sensitivity analyses

Sensitivity analyses examining relations between child height (cm) at the 24-month assessment and sUA concentrations and trajectory groupings showed similar findings as those reported above. There were no statistically significant correlations nor adjusted associations between child height (cm) and sUA concentrations at the 24-month assessment (*n*'s = 290 and 254, respectively), and height at the 24-month assessment was not statistically different between children in the Stable vs. Increasing sUA groups (*n *= 213 for this analysis).

Adjusted linear regression models employing MI to address missingness in the child- and family-level independent variables revealed similar findings as those from the complete case models presented above. Notable differences, detailed below, include changes in the statistically significant covariates of sUA levels at the 6-, 90-, and 154-month assessments and a weakening of the adjusted relation between child height-for-age percentile scores and sUA levels at the 90-month assessment.

#### 3.4.1. Covariates of salivary uric acid concentrations

In the adjusted linear regression model for sUA concentrations at the 6-month assessment using MI, birthweight was positively associated with sUA levels (*b *= 0.007, robust *SE *= 0.003, *p *< 0.05, 95% CI [0.002, 0.013]) while this relation was only marginally statistically significant in the complete case regression analysis (*p *= 0.051; Supplementary Table S3). Also, the effect of family income-to-needs ratio on sUA concentrations at the 6-month assessment was statistically significant in the complete case model (Supplementary Table S3), but only marginally significant in the model employing MI (*b *= −0.06, robust *SE *= 0.03, *p *= 0.06, 95% CI [−0.13, 0.001]). The adjusted effect of age on sUA concentrations at the 90-month assessment was no longer statistically significant when the linear regression model employed MI. Similarly, the adjusted effect of child race on sUA concentrations at the 154-month assessment was only marginally statistically significant (*b *= −0.29, robust *SE *= 0.15, *p *= 0.06, 95% CI [−0.58, 0.01]) when MI was employed.

#### 3.4.2. Salivary uric acid concentrations and child weight, height, and body mass index

In adjusted linear regression models employing MI to address missingness in the child- and family-level independent variables, the strength of the association between child height-for-age percentile scores and sUA concentrations at the 90-month assessment was no longer robust to Bonferroni-corrected *α*-levels (*b *= 0.004, robust *SE *= 0.002, *p *< 0.05, 95% CI [0.0008, 0.007]).

## 4. Discussion

This study provides novel information about the assessment and characteristics of sUA from infancy to early adolescence. Importantly, many of our key findings, including positive associations between sUA concentrations and measures of child weight, height, and BMI, mirror those from investigations of serum UA in child/adolescent samples (21, 22, 24, 26, 28, 49). Consistencies in UA findings across biofluids open up new opportunities to study and monitor obesity and cardiometabolic risks using minimally-invasive, saliva-based measures on a large-scale and across the lifespan.

### 4.1. Salivary uric acid by sex and race

Consistent with results from serum studies, male and female children in our sample exhibited similar levels of sUA at all timepoints. In serum UA studies, differences in UA concentrations by sex emerge around the onset of puberty [e.g., age 12–13 years; (20, 21, 24, 50–52)] and may be related to steep increases in UA among males driven by changes in muscle mass and sex hormones (50, 53). While we expected to find sex differences in sUA concentrations at older ages in our sample, additional studies that follow children through later adolescence are needed to better assess change during this developmental stage. Future research assessing additional biopsychosocial factors associated with race and health, especially during the earliest years of life, are also needed to better understand the differences in sUA by race observed in our study. Differences in sUA concentrations between non-White and White children in our sample were not stable in magnitude nor direction across development, and they were somewhat sensitive to missing data in our sample, introducing the possibility that other, unmeasured, factors may be influencing sUA differences by race.

### 4.2. Changes in salivary uric acid concentrations across development and associations with weight, height, and body mass index

Developmental increases in serum UA have been documented and associated with changes in growth, pubertal development, sex hormones, and body composition (20, 50, 53). While the majority of children in our sample showed no significant changes in sUA levels from infancy to early adolescence, about 30% of children exhibited increasing sUA levels across childhood with steep increases from childhood to early-adolescence. This finding, along with our finding that age is positively associated with sUA levels at the 154-month assessment, is consistent with studies of serum UA that show marked increases in UA at puberty (20, 50). Also consistent with serum findings (51), we found preliminary evidence of an age-related decline in sUA concentrations during infancy/toddlerhood, especially among children in the Increasing sUA developmental trajectory group. However, there is limited research examining UA in this age group, and further investigation is needed to understand the developmental and clinical significance of this decrease in sUA concentrations during early development.

Importantly, children in the Increasing sUA group showed higher levels of sUA at all assessments, beginning in infancy. Starting around age 2, they also showed higher weight and BMI than children in the Stable sUA group. This suggests the possibility that sUA during infancy may be one potential indicator of pediatric obesity risk. Notably, differences in measures of weight, height, and BMI between the two trajectory groups were less robust, and not statistically significant, at the 154-month assessment. This reduction in group differences may be related to normative developmental increases in UA at puberty which may make it more difficult to identify at-risk children during adolescence. Self-reported health at the 154-month assessment was, however, different between the two trajectory groups with worse health reported among adolescents in the Increasing group. While preliminary, it is tempting to speculate about associations between high sUA and obesity-related health conditions among these children. Future research should examine larger samples of children longitudinally through later adolescence and early adulthood, ideally including measures of pubertal status (53) (e.g., hormone levels and changes in growth and body composition), as well as health and disease risk to further develop our understanding of the clinical utility of sUA across development as an indicator of obesogenic risk. Doing so would also advance our understanding of whether increases in sUA across development reflect typical patterns associated with physical development during puberty or risk of obesity during adolescence and later life.

Overall, our findings highlight variability in UA across development, which may begin early in life and convey obesity-related health risks. To our knowledge, previous research has not employed the type of person-centered approach to modeling developmental trajectories of UA used in this study. This, along with our restricted follow-up period ending in early adolescence, limits our ability to directly compare our trajectory results to those of serum UA studies. Future investigations using similar statistical modeling techniques and examining a wider age range are needed to fully understand variability in UA developmental changes from infancy to adolescence along with its associations with health and disease risk. Assessment of UA in saliva facilitates this work by providing a minimally-invasive and socially acceptable approach to repeated biomarker evaluations in the youngest populations.

### 4.3. Strengths, limitations, and future directions

Our use of the FLP data allowed for the prospective evaluation of sUA in a large, diverse sample using minimally-invasive measurements and sophisticated, person-centered statistical approaches. Our findings provide key guidance for the future evaluation of sUA including preliminary reference ranges for sUA across development and the identification of potential confounds of sUA concentrations (e.g., recent eating/drinking, time of day) that should be controlled for in future investigations. Importantly, our findings also highlight the need to examine individual variability in UA developmental change as conventional regression modeling approaches may mask meaningful differences in sUA trajectories.

The subsamples used in this study, however, have limited representation of racial/ethnic groups beyond non-Hispanic/Latino White and Black/African American children. Future studies with more diverse samples of children are needed to fully understand variability in sUA levels related to race, ethnicity, and related biopsychosocial factors associated with obesity and cardiometabolic risk (1). Given the two distinct developmental trajectories of sUA identified in our sample, it will also be important for future investigations to enroll and retain larger samples during the earliest years of life to better examine sUA during this critical, yet understudied, stage and its ability to predict obesity and related comorbidities (1). Longitudinal studies with larger samples sizes could also provide additional information regarding the magnitude and significance of associations between sUA concentrations and body weight or BMI measures, particularly when assessed within sUA trajectory group. In our sample, we found no statistically significant associations between sUA levels and measures of child weight, height, or BMI when examining these correlations within the sUA developmental trajectory groups. These null findings may be due to our small sample sizes for these analyses (*n*s = 156 and 61) or reduced variability of measurements within trajectory groups. However, conducting these tests also highlights the importance of assessing patterns of developmental change in sUA within the sample, as statistically significant correlations observed across all children at a given assessment time might reflect differences between children in different sUA trajectory groups.

Given the novelty of sUA assessments in public health research, our findings also highlight several gaps in understanding that should be addressed by future studies. For example, the statistically significant covariates of sUA concentrations varied by age group in our sample and were somewhat sensitive to missing data. While most variables had very low levels of missingness (e.g., <5%), some variables, including recent eating/drinking at the 90-month assessment and height and BMI percentile scores at the 24-month assessment, had high levels of missingness. Results from our sensitivity analyses addressing missingness confirmed our main findings regarding the associations between sUA concentrations and measures of child weight, height, and BMI. However, the statistical significance of other child- and family-level covariates of sUA concentrations changed in some regression models employing MI. Findings regarding the significance of these covariates, and findings from the complete case and bivariate analyses, should be interpreted in the context of high missingness for some variables and missingness related to age for BMI and height percentile indices at the 24-month assessment. Also, some key covariates, such as diet, exercise, oral health, family health history, pubertal status, and genetic factors, were not examined. Furthermore, our findings from analyses conducted with the infant and toddler assessment data are limited by a smaller size, and these analyses do not have adequate power to detect weak correlations between sUA levels and measures of body weight, height, or BMI. Salivary determinations of UA from these first two assessments may also be confounded by the use of saliva collection swabs. While developmentally appropriate, use of collection swabs at the 6- and 24-month assessments introduces potential variability in sUA concentrations related to collection method. Importantly, research suggests that sUA determinations are not significantly affected by collection technique. However, additional studies among infant/child samples are needed to investigate the impact of swab collection on sUA levels. Similarly, to our knowledge, serum-saliva correlations for UA have only been examined among adults.

Finally, additional research is also needed to elucidate the role of UA in the development of obesity and its comorbidities. While there is evidence that UA plays a critical role in the development and maintence of cardiometabolic conditions, such as hypertension and insulin resistance in adults and adolescence (54, 55), the mechanisms underlying these associations are not fully understood. Examining whether UA is a correlate or reflection of future health risks or a mechanism driving underlying disease is essential to understanding the utility of sUA as an indicator of obesogenic risks and potential target for intervention. Future studies examining sUA among clinical samples and including evaluations by medical professionals will provide key information regarding the prognostic value of sUA. Given the existing range of intervention approaches available to reduce UA, including both pharmaceutical and lifestyle approaches (54, 56–58), understanding the role of UA in disease risk and progression early in development will be critical to future public health and clinical efforts to promote child health.

### 4.4. Implications

New clinical practice guidelines from the American Academy of Pediatrics (AAP) call for the treatment of obesity in all children ages two years and older using intensive, evidence-based approaches (1). The AAP also recommends annual BMI tracking and laboratory evaluation (e.g., assessing analytes indexing metabolism, liver function, and lipid abnormalities) starting at age two for children with obesity (1). These guidelines highlight the urgency and severity of pediatric obesity as a chronic disease with serious comorbidities and implications for lifelong health (1). While preliminary, our findings could support new research and clinical efforts stemming from these guidelines. For example, minimally-invasive assessments of UA in saliva could facilitate pediatric obesity research, particularly within the youngest age groups. There is currently limited information about effective obesity treatments for these age groups, and salivary assessments of UA open up new opportunities to study the biological factors underlying obesity and treatment mechanisms in these children (1). This is particularly important as early life obesity predicts later life obesity (1) and UA concentrations in adults have been found to predict the development of cardiometabolic risk, highlighting its potential utility in identifying at-risk individuals for intervention and prevention efforts (59). Salivary UA testing could help identify individuals who might benefit from UA lowering treatment options for reducing cardiometabolic risks typically associated with obesity (60). Salivary assessments of UA, which can occur in the home using inexpensive approaches (61–63), may also allow for long-term monitoring of children/adolescents for obesity-related health conditions, such as high blood pressure, in a minimally-invasive and ecologically valid manner. Assessments of sUA could also be added to annual BMI tracking and laboratory evaluation procedures. This long-term monitoring of obesity-related disease risk is a key aspect of AAP's comprehensive obesity treatment recommendations (1).

## 5. Conclusions

We found significant associations between sUA concentrations and indices related to obesity during childhood and adolescence. While preliminary, our findings suggest sUA during early life may help identify children at risk of obesity. If the findings of this study are supported by future investigations, they would signal new opportunities for research and the large-scale assessment, monitoring, and potential prevention and treatment of health problems associated with high UA, including obesity, among children. The findings from this study contribute critical new evidence in this emerging area of research.

## Data Availability

The raw data supporting the conclusions of this article will be made available by the authors, without undue reservation.
