# Supplementary material for: Salivary uric acid across child development and associations with weight, height, and body mass index
Source: Front Pediatr. 2023 Nov 1;11:1235143. doi: 10.3389/fped.2023.1235143 (PMC10646470; doi:10.3389/fped.2023.1235143)
Supplement: Supplementary file 1 [file Table1.docx]

Supplementary Material

Salivary uric acid across child development and associations with weight, height, and body mass index

Riis, JL^*^, Dent, AL, Silke, O, Granger, DA, on behalf of program collaborators for Environmental influences on Child Health Outcomes

*** Correspondence:** Jenna L. Riis, [jriis@illinois.edu](mailto:jriis@illinois.edu)

# Supplementary Methods

# Evaluation of recent medication use: When assessing recent medication use, survey questions varied slightly across the assessments, however, response options were “yes/no” for all assessments (6-month assessment question: “In the last 2 days (48 hours) did [the participating child] take any over-the-counter or prescription medications (including aspirin, Tylenol, Pediacare)?”; 24-month assessment question: “Is [the participating child] currently taking any kind of medication?”; 90-month assessment question: “Did [the participating child] take any over the counter or prescription medications in the last 24 hours?”; 154-month assessment question: “Did you take any over the counter or prescription medications in the last 24 hours?”). Responses to these questions were combined across assessment times to generate a single variable coding for recent medication use.

# Supplementary Figures


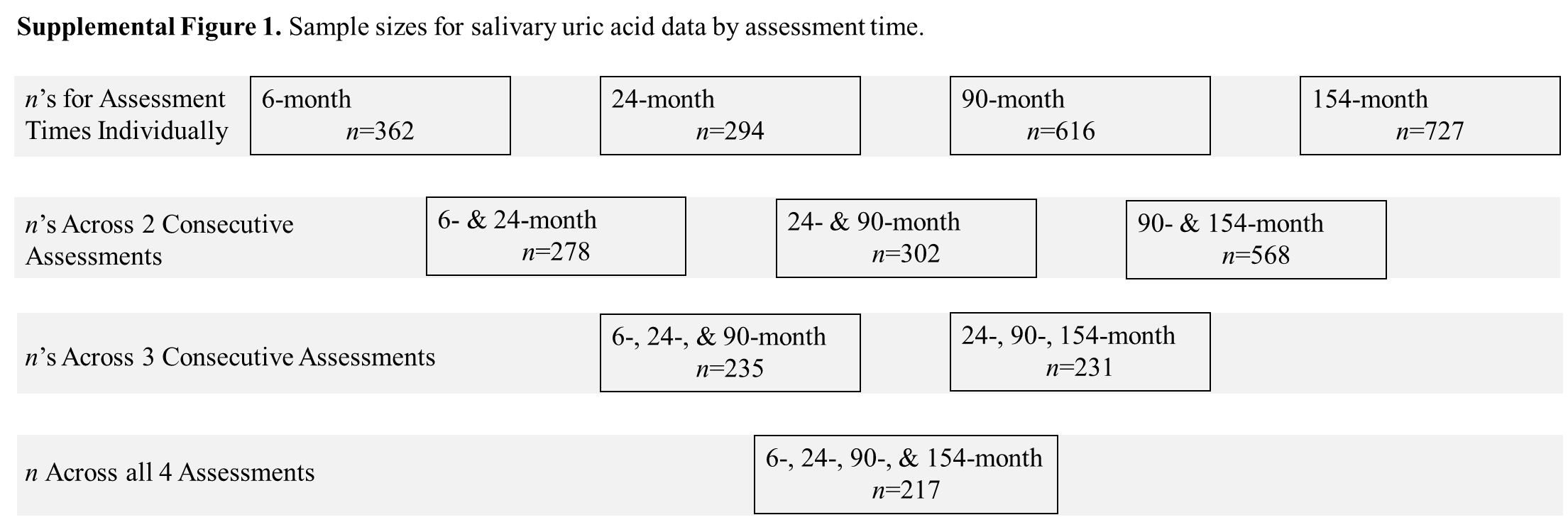


# Supplementary Tables

**Supplemental Table S1**. *Participant characteristics for assessment subsamples*.

|  | **6-month Assessment** | | | | | **24-month Assessment** | | | | | **90-month Assessment** | | | | | **154-month Assessment** | | | | |
| --- | --- | --- | --- | --- | --- | --- | --- | --- | --- | --- | --- | --- | --- | --- | --- | --- | --- | --- | --- | --- |
|  | *M* | *SD* | Min | Max | *n* | *M* | *SD* | Min | Max | *n* | *M* | *SD* | Min | Max | *n* | *M* | *SD* | Min | Max | *n* |
| Age (months) | 7.43 | 1.34 | 5.09 | 13.11 | 362 | 24.42 | 1.59 | 22.24 | 34.76 | 294 | 87.03 | 3.27 | 78.85 | 100.73 | 616 | 158.15 | 6.65 | 150.28 | 182.08 | 727 |
| Male (*n*, %) | 201 | 56% |  |  | 362 | 161 | 55% |  |  | 294 | 321 | 52% |  |  | 616 | 363 | 50% |  |  | 727 |
| Race (*n*, %) |  |  |  |  | 362 |  |  |  |  | 294 |  |  |  |  | 616 |  |  |  |  | 727 |
| White | 223 | 62% |  |  |  | 177 | 60% |  |  |  | 372 | 60% |  |  |  | 398 | 55% |  |  |  |
| Black/African American | 137 | 38% |  |  |  | 116 | 39% |  |  |  | 241 | 39% |  |  |  | 325 | 45% |  |  |  |
| American Indian or Alaska Native | 0 | 0% |  |  |  | 0 | 0% |  |  |  | 2 | <1% |  |  |  | 2 | <1% |  |  |  |
| Asian | 2 | 1% |  |  |  | 1 | <1% |  |  |  | 1 | <1% |  |  |  | 2 | <1% |  |  |  |
| Hispanic/Latino (*n*, %) | 10 | 3% |  |  | 362 | 8 | 3% |  |  | 294 | 14 | 2% |  |  | 614 | 17 | 2% |  |  | 725 |
| State (*n*, %) |  |  |  |  | 362 |  |  |  |  | 294 |  |  |  |  | 616 |  |  |  |  | 727 |
| North Carolina | 173 | 48% |  |  |  | 142 | 48% |  |  |  | 291 | 47% |  |  |  | 412 | 57% |  |  |  |
| Pennsylvania | 189 | 52% |  |  |  | 152 | 52% |  |  |  | 325 | 53% |  |  |  | 315 | 43% |  |  |  |
| Family income/needs | 2.02 | 1.77 | 0.00 | 16.47 | 333 | 1.83 | 1.66 | 0.00 | 16.24 | 280 | 1.99 | 1.63 | 0 | 11.18 | 587 | 1.90 | 1.72 | 0.00 | 17.10 | 723 |
| Caregiver married (*n*, %) | 187 | 52% |  |  | 362 | 143 | 49% |  |  | 294 | 308 | 51% |  |  | 607 | 332 | 47% |  |  | 710 |
| Caregiver education (years) | 14.66 | 2.64 | 8 | 22 | 362 | 14.66 | 2.64 | 8 | 22 | 294 | 14.60 | 2.88 | 7 | 22 | 607 | 14.45 | 2.82 | 7.00 | 22.00 | 710 |
| Weight-for-age percentile | 56.75 | 28.06 | 0.33 | 99.98 | 360 | 57.79 | 29.95 | 0.09 | 100.00 | 292 | 64.82 | 29.38 | 0.01 | 99.99 | 612 | 72.25 | 27.68 | 0.00 | 99.99 | 725 |
| Height-for-age percentile | 53.91 | 28.81 | 0.00 | 99.98 | 361 | 50.42 | 27.82 | 0.90 | 99.67 | 218 | 57.21 | 29.77 | 0.11 | 100.00 | 609 | 57.42 | 29.97 | 0.09 | 100.00 | 723 |
| BMI-for-age percentile |  |  |  |  |  | 64.93 | 26.83 | 0.00 | 99.84 | 143 | 65.61 | 28.14 | 0.00 | 99.82 | 606 | 72.52 | 27.30 | 0.00 | 99.78 | 723 |
| Current health (*n*, %) |  |  |  |  | 362 |  |  |  |  |  |  |  |  |  | 614 |  |  |  |  | 726 |
| Excellent | 190 | 52% |  |  |  |  |  |  |  |  | 371 | 60% |  |  |  | 137 | 19% |  |  |  |
| Very good | 88 | 24% |  |  |  |  |  |  |  |  | 139 | 23% |  |  |  | 190 | 26% |  |  |  |
| Good | 54 | 15% |  |  |  |  |  |  |  |  | 80 | 13% |  |  |  | 301 | 41% |  |  |  |
| Fair | 25 | 7% |  |  |  |  |  |  |  |  | 21 | 3% |  |  |  | 86 | 12% |  |  |  |
| Poor | 5 | 1% |  |  |  |  |  |  |  |  | 3 | <1% |  |  |  | 12 | 2% |  |  |  |

*Notes*: Samples overlap across assessments. See text and Supplemental Figure 1 for information about the degree of overlap and follow-up across assessments. Caregiver marital status and education were self-reported at the 6-month visit. Current child health was reported by caregivers at the 6- and 90-month assessments and self-reported at the 154-month assessment (data are not available at the 24-month assessment). Child height and weight were measured by study staff. BMI= body mass index; *M*= mean; *SD*= standard deviation; Min= minimum; Max= maximum.

**Supplemental Table S2.** *Salivary uric acid concentrations (mg/dL) from infancy to early adolescence among healthy children by race category.*

|  | **Assessment Time** | | | | | | | |
| --- | --- | --- | --- | --- | --- | --- | --- | --- |
|  | **6-months*** | | **24-months** | | **90-months** | | **154-months*** | |
|  | **White** | **non-White** | **White** | **non-White** | **White** | **non-White** | **White** | **non-White** |
| Mean | 1.49 | 1.30 | 1.53 | 1.47 | 1.39 | 1.32 | 2.37 | 2.60 |
| *SD* | 0.97 | 1.08 | 1.01 | 0.85 | 1.34 | 1.16 | 2.07 | 1.76 |
| Median | 1.47 | 1.00 | 1.33 | 1.34 | 1.13 | 1.19 | 2.14 | 2.44 |
| Min | 0.04 | 0.04 | 0.04 | 0.04 | 0.04 | 0.04 | 0.04 | 0.04 |
| Max | 4.87 | 6.84 | 4.86 | 4.60 | 8.33 | 5.94 | 13.20 | 10.33 |
| *n* | 223 | 139 | 177 | 117 | 372 | 244 | 398 | 329 |

*Note:* Race was reported by primary caregivers at the 6-month assessment. Children in the non-White category include: at the 6-month assessment- 137 children characterized as Black/African American and 2 characterized as Asian; at the 24-month assessment- 116 children characterized as Black/African American and 1 characterized as Asian; at the 90-month assessment- 241 children characterized as Black/African American, 2 characterized as American Indian or Alaska Native, and 1 characterized as Asian; and at the 154-month assessment- 325 children characterized as Black/African American, 2 characterized as American Indian or Alaska Native, and 2 characterized as Asian. Samples at each assessment are overlapping. See text and Supplemental Figure 1 for information about the degree of overlap and follow-up across assessments. Concentrations of sUA that were too low to be measured by the assay were replaced with 0.035 mg/dL. *SD*=standard deviation; Min=minimum; Max=maximum. **p*<0.05 for differences in sUA concentrations across race category (see text for detailed results).

**Supplemental Table S3**. *Adjusted associations between child sociodemographic and study characteristics and salivary uric acid concentrations (mg/dL) and among healthy young children.*

|  | **6-month Assessment** | | | | | | | | **24-month Assessment** | | | | | | | | **90-month Assessment** | | | | | | | | **154-month Assessment** | | | | | | | |
| --- | --- | --- | --- | --- | --- | --- | --- | --- | --- | --- | --- | --- | --- | --- | --- | --- | --- | --- | --- | --- | --- | --- | --- | --- | --- | --- | --- | --- | --- | --- | --- | --- |
|  | *B* | *SE* | *t* | *p* | 95% CI | | R^2^ | *n* | *B* | *SE* | *t* | *p* | 95% CI | | R^2^ | *n* | *B* | *SE* | *t* | *p* | 95% CI | | R^2^ | *n* | *B* | *SE* | *t* | *p* | 95% CI | | R^2^ | *n* |
| Child age | -0.042 | 0.042 | -1.020 | 0.308 | -0.124 | 0.039 | 0.073 | 326 | 0.017 | 0.029 | 0.570 | 0.566 | -0.041 | 0.074 | 0.067 | 257 | **-0.034** | **0.017** | **-2.090** | **0.037** | **-0.067** | **-0.002** | 0.029 | 581 | **0.022** | **0.010** | **2.170** | **0.031** | **0.002** | **0.042** | 0.033 | 715 |
| Child sex | 0.016 | 0.106 | 0.150 | 0.878 | -0.193 | 0.226 |  |  | 0.132 | 0.113 | -1.170 | 0.243 | -0.090 | 0.353 |  |  | 0.021 | 0.099 | 0.210 | 0.831 | -0.174 | 0.216 |  |  | 0.122 | 0.139 | 0.880 | 0.378 | -0.150 | 0.394 |  |  |
| Time since noon | 0.000 | 0.000 | -1.050 | 0.292 | -0.001 | 0.000 |  |  | **-0.001** | **0.000** | **-3.920** | **0.000** | **-0.002** | **-0.001** |  |  | **-0.001** | **0.000** | **-2.770** | **0.006** | **-0.002** | **-0.000** |  |  | 0.000 | 0.000 | 0.490 | 0.622 | -0.001 | 0.001 |  |  |
| Family income/needs | **-0.063** | **0.031** | **-2.050** | **0.042** | **-0.123** | **-0.002** |  |  | -0.011 | 0.033 | -0.330 | 0.743 | -0.076 | 0.054 |  |  | 0.049 | 0.036 | 1.360 | 0.176 | -0.022 | 0.121 |  |  | 0.038 | 0.047 | 0.810 | 0.417 | -0.054 | 0.131 |  |  |
| Child race | **0.262** | **0.122** | **2.150** | **0.032** | **0.023** | **0.502** |  |  | 0.104 | 0.122 | 0.850 | 0.395 | -0.137 | 0.345 |  |  | 0.092 | 0.111 | 0.830 | 0.408 | -0.126 | 0.311 |  |  | **-0.300** | **0.150** | **-2.000** | **0.046** | **-0.595** | **-0.006** |  |  |
| Recently ate/drank | **-0.253** | **0.105** | **-2.420** | **0.016** | **-0.459** | **-0.047** |  |  |  | | | | | |  |  |  |  |  |  |  |  |  |  | 0.188 | 0.140 | 1.350 | 0.179 | -0.086 | 0.462 |  |  |
| Current health |  |  |  |  |  |  |  |  |  | | | | | |  |  |  |  |  |  |  |  |  |  |  |  |  |  |  |  |  |  |
| very good | -0.087 | 0.118 | -0.730 | 0.465 | -0.319 | 0.146 |  |  |  | | | | | |  |  | 0.067 | 0.127 | 0.530 | 0.597 | -0.182 | 0.316 |  |  | 0.300 | 0.210 | 1.430 | 0.153 | -0.112 | 0.712 |  |  |
| good | 0.306 | 0.177 | 1.730 | 0.085 | -0.042 | 0.654 |  |  |  | | | | | |  |  | 0.045 | 0.148 | 0.300 | 0.761 | -0.245 | 0.335 |  |  | 0.232 | 0.188 | 1.230 | 0.218 | -0.138 | 0.602 |  |  |
| fair/poor | 0.042 | 0.177 | 0.240 | 0.814 | -0.306 | 0.389 |  |  |  | | | | | |  |  | -0.281 | 0.244 | -1.150 | 0.249 | -0.759 | 0.197 |  |  | **0.839** | **0.252** | **3.320** | **0.001** | **0.343** | **1.334** |  |  |
| Birth weight | 0.006 | 0.003 | 1.960 | 0.051 | -0.000 | 0.011 |  |  |  | | | | | |  |  |  | | | | | |  |  |  | | | | | |  |  |
| Constant | **1.152** | **0.493** | **2.340** | **0.020** | **0.181** | **2.122** |  |  | 1.095 | 0.719 | 1.520 | 0.129 | -0.321 | 2.511 |  |  | **4.332** | **1.418** | **3.050** | **0.002** | **1.547** | **7.118** |  |  | -1.472 | 1.681 | -0.880 | 0.381 | -4.772 | 1.828 |  |  |

*Notes*: Samples at each assessment are overlapping. See text and Supplemental Figure 1 for information about the degree of overlap and follow-up across assessments. Cases excluded due to poor model fit at each assessment time: 6-month model: 2 cases excluded; 24-month model: 1 case excluded; 90-month model: 4 cases excluded; 154-month model- 4 cases excluded. All models were conducted with robust variance, and robust standard errors are presented. Recent eating/drinking and current health data were not available at the 24-month assessment. *SE*= standard error; CI=confidence interval. Parameters statistically significant at the *p*<0.05 level are bolded. Reference categories are: sex- female; race- non-White; current health: excellent.

**Supplemental Table S4.** *Weight, height, and body mass index (BMI) measurements among healthy children by salivary uric acid (sUA) developmental trajectory group.*

|  | | **Assessment Time** | | | | | | | |
| --- | --- | --- | --- | --- | --- | --- | --- | --- | --- |
|  |  | **6-months** | | **24-months** | | **90-months** | | **154-months** | |
|  |  | **Increasing sUA Group** | **Stable**  **sUA Group** | **Increasing sUA Group** | **Stable**  **sUA Group** | **Increasing**  **sUA Group** | **Stable**  **sUA Group** | **Increasing sUA Group** | **Stable**  **sUA Group** |
| Weight-for-age percentile | Mean (*SD*) | 61.55 (27.24) | 56.39 (27.71) | 65.16 (29.44)* | 54.69 (28.98) | 74.76 (26.77)** | 62.66 (30.25) | 79.53 (23.28)◦ | 71.48 (28.44) |
|  | *n* | 61 | 155 | 60 | 156 | 61 | 155 | 60 | 155 |
| Height-for-age percentile | Mean (*SD*) | 54.77 (29.12) | 55.34 (27.47) | 56.23 (26.41) | 49.77 (28.73) | 64.55 (27.53) | 57.53 (29.68) | 67.80 (28.51)◦ | 60.21 (30.51) |
|  | *n* | 61 | 155 | 42 | 114 | 61 | 154 | 59 | 155 |
| BMI-for-age percentile | Mean (*SD*) |  |  | 63.94 (28.29) | 61.78 (26.01) | 72.90 (27.37)* | 61.69 (29.88) | 77.39 (23.41)◦ | 70.39 (28.74) |
|  | *n* |  |  | 26 | 71 | 61 | 153 | 59 | 155 |

*Notes*: Salivary uric acid (sUA) developmental trajectory group membership determined via group-based trajectory modeling conducted with *n*=217. The Increasing sUA group had 61 children and the Stable sUA group had 156 children (see text and Figure 2 for detailed results). *SD*= standard deviation.***p*<0.01, **p*<0.05, ◦*p*<0.10 for differences between children in the Increasing vs. Stable sUA groups within assessment time.
